# Efficacy and safety of telaprevir, a new protease inhibitor, for difficult-to-treat patients with genotype 1 chronic hepatitis C

**DOI:** 10.1111/j.1365-2893.2011.01528.x

**Published:** 2012-02

**Authors:** N Hayashi, T Okanoue, H Tsubouchi, J Toyota, K Chayama, H Kumada

**Affiliations:** 1 Kansai-Rosai HospitalHyogo, Japan; 2 Department of Gastroenterology and Hepatology, Saiseikai Suita HospitalOsaka, Japan; 3 Department of Digestive and Life-style Related Disease, Kagoshima University Graduate School of Medical and Dental SciencesKagoshima, Japan; 4 Department of Gastroenterology, Sapporo Kosei General HospitalHokkaido, Japan; 5 Department of Medical and Molecular Science, Division of Frontier Medical Science, Programs for Biomedical Research, Graduate School of Biomedical Science, Hiroshima UniversityHiroshima, Japan;; 6 Department of Hepatology, Toranomon HospitalTokyo, Japan

**Keywords:** direct-acting antiviral, peginterferon, ribavirin, sustained virological response, treatment failure

## Abstract

The aims of this phase III study were to assess the efficacy and safety of telaprevir in combination with peginterferon alfa-2b (PEG-IFN) and ribavirin (RBV) for difficult-to-treat patients who had not achieved sustained virological response (SVR) to prior regimens in Japan. The subjects were 109 relapsers (median age of 57.0 years) and 32 nonresponders (median age of 57.5 years) with hepatitis C virus genotype 1. Patients received telaprevir (750 mg every 8 h) for 12 weeks and PEG-IFN/RBV for 24 weeks. The SVR rates for relapsers and nonresponders were 88.1% (96/109) and 34.4% (11/32), respectively. Specified dose modifications of RBV that differed from that for the standard of care were introduced to alleviate anaemia. RBV dose reductions were used for 139 of the 141 patients. The SVR rates for relapsers did not depend on RBV dose reduction for 20–100% of the planned dose (SVR rates 87.5–100%, *P* < 0.05). Skin disorders were observed in 82.3% (116/141). Most of the skin disorders were controllable by anti-histamine and/or steroid ointments. The ratios of discontinuation of telaprevir only or of all the study drugs because of adverse events were 21.3% (30/141) and 16.3% (23/141), respectively. A frequent adverse event leading to discontinuation was anaemia. Telaprevir in combination with PEG-IFN/RBV led to a high SVR rate for relapsers and may offer a potential new therapy for nonresponders even with a shorter treatment period.

## Introduction

Hepatitis C virus (HCV) affects approximately 170 million people worldwide [1]; patients with chronic hepatitis C (CHC) eventually develop cirrhosis and hepatocellular carcinoma (HCC) [2,3]. The standard of care (SOC) with peginterferon plus ribavirin (RBV) for 48 weeks is most effective for eradicating HCV genotype 1 [4], which is a dominant genotype for CHC [1]. However, the sustained virological response (SVR) rate of SOC for the treatment of naïve patients with genotype 1 is approximately <50% [5,6]. The retreatment regimen for patients who do not achieve SVR is limited to exposure to peginterferon plus RBV with modification of dose and treatment duration. Some studies have been conducted to estimate the effectiveness of peginterferon plus RBV for 48 weeks for nonresponders to prior interferon-based combination therapy, and the SVR rates in most studies did not exceed 20% [7–9]. A large randomized study of patients who had not responded to previous treatment with peginterferon alfa-2b (PEG-IFN) plus RBV gave SVR rates for peginterferon alfa-2a 180 μg/kg plus RBV for 72 weeks that were not as high as those for 48 weeks (14%, 9%) [10]. HCV patients who had failed to achieve SVR with the combination therapy displayed high risk rates of decompensated cirrhosis, HCC and liver-related mortality [11]. Therefore, it is very important to establish new regimens to increase the SVR rate and shorten the treatment period for patients who do not achieve SVR with prior treatments.

Telaprevir, classified as a direct-acting antiviral agent, is a reversible, selective, orally bioavailable inhibitor of the nonstructural NS3/4A HCV serine protease [12]. Two phase II studies (PROVE 1 and PROVE 2) on the treatment of naïve patients with genotype 1 were conducted to assess the efficacy of telaprevir for 12 weeks in combination with peginterferon and RBV for 24 weeks [13,14]. These studies demonstrated that the SVR rates of the telaprevir regimen were significantly higher compared with SOC (PROVE 1: 61%*vs* 41%, *P* = 0.02, PROVE 2: 69%*vs* 46%, *P* = 0.004). A subsequent phase II study (PROVE 3) for treatment-failure patients with genotype 1 gave SVR rates for nonresponders, relapsers and breakthroughs in the telaprevir regimen of 39%, 69% and 57%, respectively [9].

In Japan, a phase III study was conducted for the treatment of naïve patients with genotype 1 to compare the efficacy and safety between the telaprevir regimen and SOC. It has demonstrated that the SVR rate for the telaprevir regimen was significantly higher than that for SOC (73.0%*vs* 49.2%, *P* = 0.0020) [15]. We decided to conduct a phase III study to assess the efficacy and safety of telaprevir in combination with PEG-IFN and RBV in relapsers and nonresponders who had not achieved SVR to a previously administered IFN-based regimen in Japan.

## Patients and methods

### Study patients

Relapsers and nonresponders were enrolled in Study 1 (ClinicalTrials.gov Identifier: NCT00780910) and Study 2 (ClinicalTrials.gov Identifier: NCT00781274), respectively. Relapsers were defined as patients who had been previously treated for CHC and had undetectable HCV RNA during interferon or peginterferon therapy (including combination with RBV). Nonresponders were defined as patients who were previously treated for CHC and had never had undetectable HCV RNA for more than 24 weeks with interferon or peginterferon therapy (including combination with RBV).

The patients were enrolled from 17 sites in Japan. Patients considered eligible were of 20–65 years of age, had CHC because of HCV genotype 1 (defined by NS5B sequence) [16] and ≥5.0 log_10_ IU/mL HCV RNA level at the screening test, had been previously treated for CHC with interferon or peginterferon therapy (including combination with RBV), had a body weight of 40 kg or more and below 120 kg, could be hospitalized for at least 2 weeks after the first administration, were not pregnant and agreed to contraception from the screening period to 24 weeks after the last dosing of the study drug. The patients were excluded if they had a haemoglobin level of <12 g/dL, neutrophil count of <1500/mm^3^, platelet count of <100 000/mm^3^, were positive for HBs antigen and HIV antibodies at the screening test, had chronic renal failure or creatinine clearance of ≤50 mL/min, depression, schizophrenia or its history, history of suicide attempt, decompensated cirrhosis, previous or current HCC or other malignancies, autoimmune hepatitis, alcoholic liver disease or haemochromatosis.

All patients provided written informed consent before participating in the study. These studies were approved by each site’s institutional review board and conducted in accordance with good clinical practice and the Declaration of Helsinki.

### Study design

All patients received PEG-IFN (PegIntron®; MSD, Tokyo, Japan) at a dose of 1.5 μg/kg per week subcutaneously, RBV (Rebetol®; MSD) at a dose of 600 mg per day (for body weight ≤60 kg), 800 mg per day (for body weight >60 to ≤80 kg) or 1000 mg per day (for body weight >80 kg) and telaprevir (MP-424; Mitsubishi Tanabe Pharma, Osaka, Japan) at a dose of 750 mg every 8 h after food. The patients were treated with telaprevir, PEG-IFN and RBV for 12 weeks, followed by PEG-IFN and RBV (PEG-IFN/RBV) for 12 weeks. All patients had a 24-week follow-up period after the last dosing of study drugs to assess SVR.

### Dose modification of study drugs

Specified dose modification of RBV that differed from the dose for SOC was introduced to alleviate anaemia. The initial dose of RBV was reduced by 200 mg per day in case of a haemoglobin level <13 g/dL at baseline. The RBV dose was reduced by 200 mg per day in patients receiving 600 or 800 mg per day (by 400 mg per day in those receiving 1000 mg) when the haemoglobin level was <12 g/dL and was reduced by an additional 200 mg per day when the haemoglobin level was <10 g/dL. The RBV dose was also reduced by 200 mg per day if the haemoglobin level dropped ≥1 g/dL within 1 week, and this level was <13 g/dL. Telaprevir was withdrawn when the haemoglobin level was <8.5 g/dL. PEG-IFN/RBV were withdrawn or interrupted when the haemoglobin level was <8.5 g/dL. The dose modifications of PEG-IFN were followed by SOC. Dose modification and interruption of telaprevir were not allowed. Telaprevir was withdrawn if serious adverse events appeared. The use of erythropoietin was not allowed for elevating the haemoglobin level.

### Stopping rules

Patients could be discontinued from the study at any time if the investigator or sponsor determined that it was not in the interest of the patient to continue the study or the patient wished to withdraw from the study. The study drugs were discontinued if the patients had a haemoglobin level of <8.5 g/dL, white blood cell count of <1000/mm^3^, neutrophil count of <500/mm^3^ or platelet count of <50 000/mm^3^.

In case of the following criteria for serum HCV RNA viral kinetics measured during the treatment period, discontinuation of the study drugs was decided at the investigator’s discretion. (i) When the following criteria applied twice consecutively: (a) the amount of change from the lowest value for HCV RNA level exceeded 2.0 log_10_ IU/mL and (b) HCV RNA level exceeded 2.0 log_10_ IU/mL after it had been confirmed to be <1.2 log_10_ IU/mL. (ii) When the serum HCV RNA level at 13 weeks after administration of study drugs did not decrease by >2.0 log_10_ IU/mL from the baseline level.

### Efficacy assessments

Serum HCV RNA levels were measured using the COBAS TaqMan HCV test (Roche Diagnostics Co. Ltd., Tokyo, Japan). The linear dynamic range was 1.2–7.8 log_10_ IU/mL. Samples with undetectable HCV RNA were reported as ‘<1.2 log_10_ IU/mL (no detectable HCV RNA)’. Measurements were obtained at week 4 before day 1 of the screening period: at days 1 (predose), 2 and 3; weeks 1, 2, 4, 6, 8, 10, 12, 14, 16, 18, 20, 22 and 24 of the treatment period; and weeks 2, 4, 8, 12, 16, 20 and 24 of the follow-up period.

The primary endpoint was a SVR defined as an undetectable HCV RNA level 24 weeks after the end of treatment. Relapse, breakthrough, and nonresponse were defined based on AASLD Guidelines as follows [4]: ‘relapse’ was a state of undetectable serum HCV RNA at the end of treatment and reappearance of serum HCV RNA during the follow-up period; ‘breakthrough’ was a state of undetectable serum HCV RNA and reappearance of serum HCV RNA during the treatment period; and ‘nonresponse’ was a state of continuously detectable serum HCV RNA during the treatment period.

### Safety assessments

All adverse events were recorded up to the last visit and coded using MedDRA/J version 13.0. (MedDRA Japanese Maintenance Organization, Tokyo, Japan) Measurements for chemical laboratory data were obtained at week 4 before day 1 of the screening period: at day 1 (predose); weeks 1, 2, 4, 8, 10, 12, 14, 16, 18, 20 and 24 of the treatment period; and weeks 2, 4, 8, 12 and 24 of the follow-up period. Electrocardiogram (ECG) and fundus examinations were performed once during the screening period. Adverse events, haematological and chemical laboratory data, and vital signs were assessed and summarized. The severity of rash was categorized into three grades.

### Statistical analysis

Sustained virological response rates were evaluated for the full analysis set. Categorical variables were compared by Fisher’s exact test. Statistical analyses were performed using the statistical software SAS Version 9.1 (SAS Institute Inc., Cary, NC, USA), and a *P* value < 0.05 was considered significant.

## Results

### Study patients

From November 2008 to August 2009, a total of 168 patients [Study 1 (*N* = 135) and Study 2 (*N* = 33)] were screened, and 141 patients [Study 1 (*N* = 109) and Study 2 (*N* = 32)] received at least one dose of a study drug. The baseline characteristics of the study patients are shown in Table 1. Patients previously treated with PEG-IFN (with or without RBV) and IFN (with or without RBV) in Study 1 and Study 2 accounted for 75.2% (82 of 109) and 24.7% (27 of 109) and 90.6% (29 of 32) and 9.4% (3 of 32), respectively. The median of age, weight, haemoglobin level, platelet count and HCV RNA level for Study 1 and Study 2 were 57.0 and 57.5 years, 62.5 and 61.3 kg, 14.7 and 14.5 g/dL, 17.8 and 17.85 × 10^4^/mm^3^, and 6.75 and 6.78 log_10_ IU/mL, respectively. Patients over 50 years of age accounted for 81.7% (89 of 109) and 81.3% (26 of 32), respectively.

**Table 1:** Baseline characteristics of study patients

	Study 1 (relapsers) *N *= 109	Study 2 (nonresponders) *N *= 32
Gender –*n* (%)		
Men	66 (60.6)	17 (53.1)
Women	43 (39.4)	15 (46.9)
Age, years – median (range)	57.0 (20, 65)	57.5 (40, 65)
Weight, kg – median (range)	62.50 (41.0, 92.5)	61.30 (44.9, 92.5)
BMI, kg/m^2^– median (range)*	23.10 (18.0, 32.4)	22.60 (17.1, 31.2)
ALT (IU/L) – median (range)†	36.0 (16, 302)	48.0 (17, 190)
Haemoglobin (g/dL) – median (range)	14.70 (12.0, 17.8)	14.50 (12.3, 16.6)
White blood cell count (/mm^3^)	4680.0 (2490, 15940)	4830.0 (3040, 8000)
Platelet count (×10^4^/mm^3^) – median (range)	17.80 (9.9, 33.8)	17.85 (9.1, 26.2)
HCV RNA (log_10_ IU/mL) – median (range)‡	6.75 (5.2, 7.6)	6.78 (6.0, 7.7)
HCV genotype 1 subtype –*n* (%)		
1a	0 (0.0)	1 (3.1)
1b	109 (100.0)	31 (96.9)
Prior therapy for chronic hepatitis C –*n* (%)		
Interferon	13 (11.9)	1 (3.1)
Interferon plus ribavirin	14 (12.8)	2 (6.3)
Peginterferon	3 (2.8)	0 (0.0)
Peginterferon plus ribavirin	79 (72.5)	29 (90.6)

HCV, hepatitis C virus.

* The body mass index (BMI) is the weight in kilograms divided by the square of the height in metres

† Alanine aminotransferase

‡ The HCV RNA level was measured using the COBAS TaqMan HCV test (Roche).

### Efficacy in study 1 (relapsers)

**Fig 1:**
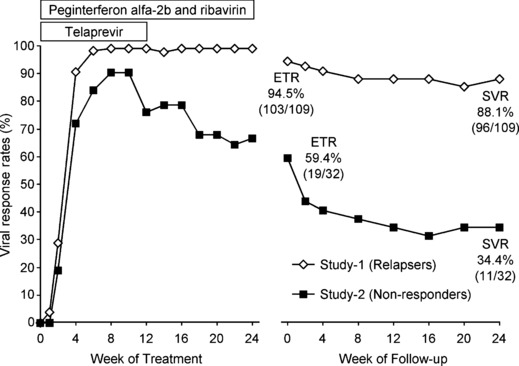
Undetectable hepatitis C virus RNA rates at each measurement point. SVR, sustained virological response; ETR, end-of-treatment response.

**Fig 2:**
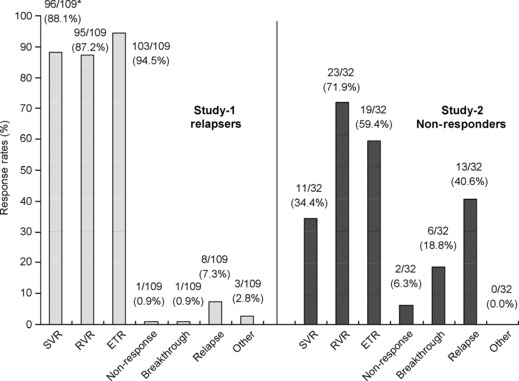
Response rates of patients with virological response. *Number of patients who achieved SVR in each subgroup/*N* (%). SVR, sustained virological response; RVR, rapid viral response; ETR, end-of-treatment response.

Figure 1 shows the change in the undetectable HCV RNA rates at each measurement point. The rapid viral response (RVR) rate and the end of treatment response (ETR) rate were 87.2% (95/109) and 94.5% (103/109), respectively. The SVR rate, nonresponse, breakthrough and relapse were 88.1% (96/109), 0.9% (1/109), 0.9% (1/109) and 7.3% (8/109), respectively (Fig. 2).

**Fig 3:**
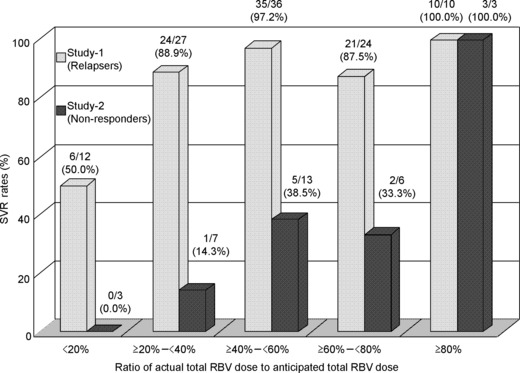
Sustained virological response rates according to adherence to the ribavirin dose.

Factors influencing the SVR rate are compared in Table 2. The SVR rate in the patients who achieved undetectable HCV RNA at ≤week 4 was significantly higher than that in the patients who achieved undetectable HCV RNA at >week 4 (91.8%*vs* 66.7%, *P* = 0.0487). Also, the SVR rate for men was significantly higher than that for women (93.9%*vs* 79.1%, *P* = 0.0316). The SVR rate with discontinuation of all the study drugs was significantly lower than that with discontinuation of only telaprevir or no discontinuation of the study drugs (all the study drugs: 60.0%, only telaprevir: 95.0% and no discontinuation: 94.2%, *P* = 0.0007). In contrast, there was no difference in the SVR rate in relation to HCV RNA level and prior therapy for CHC. SVR rates by the ratio of the actual total RBV dose to the anticipated total RBV dose were evaluated (Fig. 3). The SVR rates did not depend on RBV dose reduction for 20–100% of the planned dose (87.5–100%, *P* < 0.05).

**Table 2:** SVR rates stratified by demographic, undetectable HCV RNA and discontinuation of study drug treatment

	Study 1 (relapsers) *N *= 109	Study 2 (nonresponders) *N *= 32
Gender –*n*/*N* (%)		
Male	62/66 (93.9)	8/17 (47.1)
Female	34/43 (79.1)	3/15 (20.0)
*P*-value	0.0316	0.1475
Age –*n*/*N* (%)		
≤49	18/20 (90.0)	2/6 (33.3)
≥50	78/89 (87.6)	9/26 (34.6)
*P*-value	1.0000	1.0000
HCV RNA (log_10_ IU/mL) –*n*/*N* (%)		
≥7.0	26/30 (86.7)	5/10 (50.0)
<7.0	70/79 (88.6)	6/22 (27.3)
*P*-value	0.7498	0.2515
Prior therapy for chronic hepatitis C –*n*/*N* (%)		
Interferon	12/13 (92.3)	1/1 (100.0)
Interferon plus ribavirin	13/14 (92.9)	2/2 (100.0)
Peginterferon	3/3 (100.0)	– (–)
Peginterferon plus ribavirin	68/79 (86.1)	8/29 (27.6)
*P*-value	0.9271	0.0333
Undetectable –*n*/*N* (%)		
≤Week 4	90/98 (91.8)	9/23 (39.1)
>Week 4 ≤end of treatment	6/9 (66.7)	2/7 (28.6)
*P*-value	0.0487	1.0000
Discontinuation of study drug treatment –*n*/*N* (%)		
No discontinuation	65/69 (94.2)	9/20 (45.0)
Telaprevir only	19/20 (95.0)	2/7 (28.6)
All study drugs	12/20 (60.0)	0/5 (0.0)
*P*-value	0.0007	0.1711

SVR, sustained virological response; HCV, hepatitis C virus.

SVR was defined as an undetectable HCV RNA level 24 weeks after the end of treatment.

### Efficacy in study 2 (nonresponders)

The RVR and ETR rates were 71.9% (23/32) and 59.4% (19/32), respectively (Fig. 1). The SVR rate, nonresponse, breakthrough and relapse were 34.4% (11/32), 6.3% (2/32), 18.8% (6/32) and 40.6% (13/32), respectively (Fig. 2). There was no difference in the SVR rate in relation to baseline characteristics, HCV RNA level and prior treatment for CHC. The SVR rates for the patients who received 40–80% RBV dose reduction were over 30% (Fig. 3).

### Safety

Adverse events were observed in all the patients in Study 1 and Study 2. Adverse events observed in at least 15% of the patients in each clinical study are listed in Table 3. Adverse events were similar between Study 1 and Study 2. Most of the adverse events were mild and moderate. Serious adverse events in Study 1 and Study 2 were reported in 11.9% (13/109) and 9.4% (3/32) of the patients, respectively. The ratios of discontinuation of all the study drugs because of adverse events in Study 1 and Study 2 were 17.4% (19/109) and 12.5% (4/32), respectively. A frequent adverse event leading to discontinuation was anaemia. Discontinuation rates of all the study drugs because of anaemia in Study 1 and Study 2 were 10.1% (11/109) and 9.4% (3/32), respectively. One death was reported in Study 1. One patient in Study 1 died of pulmonary embolism. Causality of PEG-IFN and RBV was classified as ‘probably related’ and that of telaprevir was classified as ‘possibly related’.

**Table 3:** Most common adverse events

MedDRA/J (Version.13.0) preferred term –*n* (%)	Study 1 (relapsers) *N *= 109	Study 2 (nonresponders) *N *= 32	Total *N *= 141
Anaemia	96 (88.1)	32 (100.0)	128 (90.8)
Pyrexia	90 (82.6)	30 (93.8)	120 (85.1)
White blood cell count decreased	83 (76.1)	22 (68.8)	105 (74.5)
Blood uric acid increased	72 (66.1)	25 (78.1)	97 (68.8)
Platelet count decreased	73 (67.0)	22 (68.8)	95 (67.4)
Malaise	60 (55.0)	23 (71.9)	83 (58.9)
Decreased appetite	56 (51.4)	15 (46.9)	71 (50.4)
Hyaluronic acid increased	56 (51.4)	15 (46.9)	71 (50.4)
Rash	39 (35.8)	16 (50.0)	55 (39.0)
Headache	42 (38.5)	10 (31.3)	52 (36.9)
Blood creatinine increased	36 (33.0)	12 (37.5)	48 (34.0)
Insomnia	34 (31.2)	11 (34.4)	45 (31.9)
Blood bilirubin increased	34 (31.2)	10 (31.3)	44 (31.2)
Alopecia	35 (32.1)	7 (21.9)	42 (29.8)
Diarrhoea	31 (28.4)	7 (21.9)	38 (27.0)
Dysgeusia	29 (26.6)	6 (18.8)	35 (24.8)
Vomiting	26 (23.9)	8 (25.0)	34 (24.1)
Drug eruption	24 (22.0)	10 (31.3)	34 (24.1)
Nausea	24 (22.0)	4 (12.5)	28 (19.9)
Abdominal discomfort	22 (20.2)	6 (18.8)	28 (19.9)
Blood triglycerides increased	19 (17.4)	8 (25.0)	27 (19.1)
Pruritus	20 (18.3)	2 (6.3)	22 (15.6)
Arthralgia	18 (16.5)	4 (12.5)	22 (15.6)
Nasopharyngitis	19 (17.4)	2 (6.3)	21 (14.9)
Stomatitis	13 (11.9)	6 (18.8)	19 (13.5)
Back pain	12 (11.0)	5 (15.6)	17 (12.1)
Blood phosphorus decreased	10 (9.2)	6 (18.8)	16 (11.3)

The adverse events listed are those that were reported in at least 15% of patients in each clinical study.

Adverse events related to skin disorders were observed in 82.3% (116/141) of the patients. Skin disorders reported in over 10% of the patients were rash in 39.0% (55/141), drug eruption in 24.1% (34/141), injection site reaction in 12.8% (18/141) and injection site erythema in 12.8% (18/141) of the patients. Most of the skin disorders were controllable by anti-histamine and/or steroid ointments. Grade 3 (severe) skin disorders in Study 1 and Study 2 were reported in 6.4% (7/109) and 6.3% (2/32) of the patients, respectively. Discontinuation of all the study drugs because of skin disorders in Study 1 amounted to 3.7% (4/109). No discontinuation because of skin disorders occurred in Study 2.

**Fig 4:**
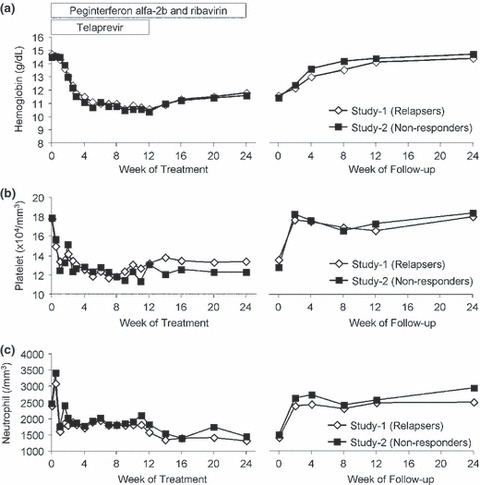
Changes in hematology parameters. Median haemoglobin levels (a), median platelet counts (b) and median neutrophil counts (c) were plotted during treatment and follow-up periods.

Figure 4 shows the changes in haemoglobin levels, platelet counts and neutrophil counts during the treatment and follow-up periods. Changes in the haematological parameters were similar between Study 1 and Study 2. The platelet count and neutrophil count decreased sharply within 4 weeks and then gradually decreased. Despite the modification of RBV, the median haemoglobin levels in Study 1 and Study 2 decreased to 10.6 and 10.4 g/dL at week 12, respectively. No patient discontinued all the study drugs because of neutrophil decrease. The haematological parameters recovered to the baseline level at the end of the follow-up period.

## Discussion

This phase III study was planned and conducted to assess the efficacy and safety of telaprevir in combination with PEG-IFN/RBV for relapsers and nonresponders. Most of the patients who participated in this study had received a prior PEG-IFN/RBV regimen. Despite a shorter treatment period, the SVR rates for relapsers and nonresponders were 88.1% and 34.4%, respectively. The result indicates that the HCV RNA response to previous treatment history should be one of the diagnostic factors for predicting SVR.

The SVR rate for men was significantly higher than that for women in the relapser group (93.9%*vs* 79.1%, *P* = 0.0316). There was no significant difference in other characteristics of the patients in that group. Once the relapsers had achieved undetectable HCV RNA, this condition was sustained until the end of the treatment period. The patients who achieved RVR had a higher SVR rate than the patients who had no RVR in the relapser group (91.8%*vs* 66.7%, *P* = 0.0487).

In contrast, there was no significant difference related to characteristics in the nonresponder group. The SVR rates between men and women and undetectable HCV RNA were, however, slightly different. As Study 2 for the nonresponders was of a small scale, it will be necessary to evaluate a larger number of patients. The breakthrough ratio in the nonresponders during the PEG-IFN/RBV treatment period and relapse ratio were 18.8% and 40.6%, respectively. Two patients were nonresponders with high telaprevir-resistant variants; one was subtype 1a and the only patient with this characteristic in the study.

Triple therapy for 12 weeks, followed by PEG-IFN/RBV for 12 weeks for the relapsers led to a high SVR rate. In contrast to the relapsers, all breakthroughs were observed in 18.8% of nonresponder patients after the end of telaprevir treatment, and relapse were observed in 40.6% of nonresponder patients after the end of treatment period. Continuation of telaprevir over 12 weeks and PEG-IFN/RBV over 24 weeks might be needed to achieve a higher SVR rate for nonresponders.

Dose modification of RBV that differed from that for SOC was introduced to prevent anaemia in the patients [17]. Dose reductions of RBV were observed in 98.6% of the patients, and those who had 200 mg RBV per day as a minimum dose and those who discontinued it accounted for 41.8% and 29.8%, respectively. The haemoglobin level recovered to the baseline level at the end of the follow-up period. As a result of dose modification, the change in the haemoglobin level in this study was similar to that in PROVE 3 [9]. Checking the haemoglobin level once a week during the treatment period is important. The SVR rates did not depend on RBV dose reduction among the relapsers who had over 20% of the anticipated total RBV dose (87.5–100%). Thus, it is important to monitor haemoglobin levels and continue RBV dosing appropriately to achieve SVR, even with a low RBV dose.

Adverse events related to skin disorder were reported by 82.3% of the subjects. Of the nine cases of severe skin disorders, seven occurred within 8 weeks. Telaprevir was likely to be related to the occurrence of the severe skin disorders. The mechanism of skin disorders is unknown. All the patients who discontinued treatment received immediate care from dermatologists and recovered eventually. Skin disorders should be carefully monitored by physicians in collaboration with dermatologists.

The relationship between the SVR rates and the difference in SNPs in gene IL28B or near IL28B has become clear [18,19]. With genetic variation in rs8099917, SVR rates of 83.8% and 27.6% were achieved for patients with genotype TT and non-TT who were treated with telaprevir in combination with PEG-IFN/RBV, respectively [20]. Also, genetic variations in gene ITPA related to haemoglobin decrease and reduction of RBV has been discussed for patients treated with PEG-IFN/RBV [21,22]. We did not evaluate IL28B and ITPA in this study. As anaemia was the most frequent adverse event leading to the discontinuation of the study drugs in the present study, it should become a valuable pharmacogenetic diagnostic tool to optimize the triple therapy.

In conclusion, this phase III study conducted in Japan demonstrated that telaprevir in combination with PEG-IFN/RBV had a high SVR rate for relapsers and shows promise as a potential therapy for nonresponders even with a short treatment period. Prolongation of telaprevir and PEG-IFN/RBV treatment should be a better option for achieving high SVR for nonresponders. As the data demonstrated convincingly that the benefits greatly outweigh the risks, telaprevir-based regimen is at the lead for the next generation of HCV therapies.

## Disclosures

None to declare.

## Appendix

The members of the phase III study were as follows: Sapporo Kosei General Hospital, Toranomon Hospital, Juntendo University Hospital, Musashino Red Cross Hospital, Toranomon Branch Hospital, University of Yamanashi Hospital, Shinshu University Hospital, Gifu Municipal Hospital, Ogaki Municipal Hospital, Nagoya University Hospital, Osaka University Hospital, Ikeda Municipal Hospital, Saiseikai Suita Hospital, Hiroshima University Hospital, Shin-Kokura Hospital, Kurume University Hospital and Kagoshima University Medical and Dental Hospital.

## Abbreviations

CHC: chronic hepatitis C
ETR: end of treatment response
HCC: hepatocellular carcinoma
HCV: hepatitis C virus
PEG-IFN: peginterferon alfa-2b
RBV: ribavirin
RVR: rapid viral response
SOC: standard of care
SVR: sustained virological response
